# Hypothalamic glucose sensing: making ends meet

**DOI:** 10.3389/fnsys.2014.00236

**Published:** 2014-12-10

**Authors:** Vanessa H. Routh, Lihong Hao, Ammy M. Santiago, Zhenyu Sheng, Chunxue Zhou

**Affiliations:** ^1^Department of Pharmacology and Physiology, New Jersey Medical School, Rutgers UniversityNewark, NJ, USA; ^2^Department of Pharmacology and Physiology and Graduate School of the Biomedical Sciences, New Jersey Medical School, Rutgers UniversityNewark, NJ, USA

**Keywords:** glucose-excited neurons, glucose-inhibited neurons, hypoglycemia, starvation, glucose homeostasis, orexin, agouti-related peptide, nitric oxide

## Abstract

The neuroendocrine system governs essential survival and homeostatic functions. For example, growth is needed for development, thermoregulation maintains optimal core temperature in a changing environment, and reproduction ensures species survival. Stress and immune responses enable an organism to overcome external and internal threats while the circadian system regulates arousal and sleep such that vegetative and active functions do not overlap. All of these functions require a significant portion of the body's energy. As the integrator of the neuroendocrine system, the hypothalamus carefully assesses the energy status of the body in order to appropriately partition resources to provide for each system without compromising the others. While doing so the hypothalamus must ensure that adequate glucose levels are preserved for brain function since glucose is the primary fuel of the brain. To this end, the hypothalamus contains specialized glucose sensing neurons which are scattered throughout the nuclei controlling distinct neuroendocrine functions. We hypothesize that these neurons play a key role in enabling the hypothalamus to partition energy to meet these peripheral survival needs without endangering the brain's glucose supply. This review will first describe the varied mechanisms underlying glucose sensing in neurons within discrete hypothalamic nuclei. We will then evaluate the way in which peripheral energy status regulates glucose sensitivity. For example, during energy deficit such as fasting specific hypothalamic glucose sensing neurons become sensitized to decreased glucose. This increases the gain of the information relay when glucose availability is a greater concern for the brain. Finally, changes in glucose sensitivity under pathological conditions (e.g., recurrent insulin-hypoglycemia, diabetes) will be addressed. The overall goal of this review is to place glucose sensing neurons within the context of hypothalamic control of neuroendocrine function.

## Introduction

Our neuroendocrine systems maintain homeostasis against changing internal and external stressors. In order to do this, it is necessary to carefully partition fuel to each system which requires energy without compromising a constant brain glucose supply. The primary responsibility for this fuel partitioning falls upon the hypothalamus. The hypothalamus integrates autonomic responses and endocrine function with behaviors, especially those concerned with the homeostatic requirements of everyday life. There are five basic physiological needs which are governed by the hypothalamus. These are the regulation of blood pressure and electrolyte composition, body temperature, energy metabolism, reproduction, and the emergency response to stress. In order to do this, the hypothalamus receives sensory information from the entire body. This input includes visceral sensory afferents as well as circulating hormones. The hypothalamus possesses internal sensory neurons which detect changes in local temperature, osmolality, electrolytes and nutrients such as glucose and free fatty acids. The hypothalamus then compares the information from this input with biological set points and adjusts an array of autonomic, endocrine and behavioral responses in order to maintain homeostasis (Oomura et al., 1969; Steffens et al., 1972, 1988; Sawchenko, 1998; Beverly et al., 2001; Lindberg et al., 2013; Valassi et al., 2014). This review will focus on the mechanisms underlying hypothalamic glucose sensing and the role of this system in glucose homeostasis.

Glucose is the primary fuel of the brain, thus it is essential to maintain adequate levels for brain function at all times. Hypothalamic glucose sensing neurons were discovered in the 1960s, and there is evidence that they play a role in maintaining cerebral glucose levels (Anand et al., 1964; Oomura et al., 1964; Levin et al., 2004; Fioramonti et al., 2010b). The hypothalamus also receives and integrates input from other central and peripheral glucose sensors (Routh et al., 2013). Interestingly, glucose sensing neurons make up subpopulations of hypothalamic nuclei involved in varied aspects of neuroendocrine function (Fioramonti et al., 2007; Gonzalez et al., 2008; Melnick et al., 2011; Roland and Moenter, 2011; Stanley et al., 2013). This suggests that glucose sensing neurons may contribute to the coordination of nutrient partitioning amongst neuroendocrine systems. However, given the need of the brain for glucose, we hypothesize that the main function of hypothalamic glucose sensing neurons is to ensure that brain needs are prioritized above those of other neuroendocrine or bodily functions.

## Glucose entry into the brain

When considering the physiological significance of brain glucose sensing, it is important to consider the regulation of glucose entry into the brain. The saturable glucose transporter isoform-1 is the primary glucose transporter at the blood brain barrier (Cardoso et al., 2010). However, the regulation of blood glucose entry is clearly more complicated than simple transport, and suggests the evidence of distinct diffusion barriers as will be discussed below (Mullier et al., 2009; Langlet et al., 2013). As a result, glucose concentrations in the brain are lower than that of the periphery. A landmark study by Silver and Erecinska in the 1990s showed that ventromedial hypothalamic (VMH) glucose levels in anesthetized rats were ~2.5 mM at baseline (~7.6 mM blood glucose due to the hyperglycemic effects of anesthesia) and 0.16 mM during hypoglycemia (~2.8 mM blood glucose). In this study, VMH glucose levels never exceeded 4.5 mM even when blood glucose was raised to 20 mM (Silver and Erecinska, 1994). Later microdialysis studies using the zero net flux technique in awake animals indicated that brain glucose levels at peripheral euglycemia (~5.5 mM) were ~1.5–2 mM, not only in the hypothalamus but the hippocampus and striatum as well (McNay and Gold, 1999; McNay et al., 2000, 2001; De Vries et al., 2003). Brain glucose varies with blood glucose, declining to ~0.7 mM after an overnight fast. During peripheral hypoglycemia, hypothalamic glucose fell to ~0.3 mM (Dunn-Meynell et al., 2009). These studies indicate that hypothalamic glucose levels range from ~0.2 to 4.5 mM as blood glucose levels vary from pathological hypoglycemia to hyperglycemia. Physiological hypothalamic glucose levels vary from ~0.7 to 2.5 mM between the fasted and fed state. This issue is important because although glucose concentration in the hypothalamus was first measured nearly 20 years ago, until fairly recently the majority of *in vitro* studies of glucose sensing neurons still used standard extracellular solutions containing 10–20 mM glucose. These glucose concentrations would even be considered analogous to diabetes when occurring in the blood. Thus, experimental conditions need to be considered when evaluating the relevance of the results obtained from studies of glucose sensing neurons.

There are several likely reasons for the resistance to studies of neurons *in vitro* using physiologically relevant glucose concentrations. One technical reason is that lower glucose concentrations were thought to be damaging to neurons *in vitro* or to cause neuronal silencing (Mobbs et al., 2001). This was reflected by the fact that until recently it was impossible to purchase stock neuronal culture media containing less than 20 mM glucose (media is now available without glucose to allow investigator control). However, for over 15 years our laboratory and others have routinely studied hypothalamic neurons in brain slices or after dissociation using ~2 mM glucose in the bathing medium with no evidence of neuronal damage or silencing (Levin et al., 1993; Routh et al., 1997; Song et al., 2001; Kang et al., 2004; Wang et al., 2004; Song and Routh, 2005, 2006; Burdakov et al., 2006; Canabal et al., 2007b; Fioramonti et al., 2007; Gonzalez et al., 2008; Cotero and Routh, 2009; Cotero et al., 2009; Murphy et al., 2009a,b; Melnick et al., 2011; Medeiros et al., 2012; Karnani et al., 2013; Vazirani et al., 2013). In fact, we find that glucose concentrations of 5 mM and higher obscure glucose sensing in some subtypes of glucose sensing neurons (Canabal et al., 2007a).

A more compelling argument for the use of higher glucose concentrations relates to brain regions near circumventricular organs. One important example is the arcuate nucleus (ARC) within the VMH which surrounds the median eminence. VMH glucose levels discussed above are often taken to reflect those within the larger ventromedial nucleus (VMN), which lies dorsal to the ARC. In contrast, since the median eminence possesses a fenestrated endothelium it was assumed that ARC glucose levels would be more similar to that of the blood than to the VMN. However, Dunn-Meynell et al., showed that ARC and VMN glucose levels do not differ (Dunn-Meynell et al., 2009). In this regard, the work of Vincent Prevot and colleagues also deserves careful consideration (Mullier et al., 2009). This group has convincingly demonstrated that differential organization and expression of the tight junction proteins within the ependymal cells which line the third ventricle and the median eminence leads to distinct patterns of diffusion from blood and cerebrospinal fluid. When blue Evans dye was injected into the blood, staining was restricted to the median eminence and did not diffuse into the ARC. In contrast, when the dye was injected into the third ventricle, staining was present in the ARC but not in the adjacent VMN. These data suggest that distinct diffusion barriers exist between the cerebrospinal fluid, circumventricular organs (e.g., median eminence) and the brain (even between adjacent brain regions). However, the physiological significance of diffusion from cerebrospinal fluid to the ARC is unclear given that the cerebrospinal fluid is thought predominantly to drain the brain. An even more intriguing observation from these investigators is that these barriers are not constant under all physiological conditions. For example, during fasting and glucoprivation the structural components of these hypothalamic diffusion barriers were altered such that ARC glucose levels significantly exceeded those in the VMN, although they did not reach blood levels. Furthermore, blood glucose levels appear to play a key role in driving these structural changes in the blood- and cerebrospinal fluid-brain barriers (Langlet et al., 2013). Together, these data indicate that while brain glucose levels are clearly lower than those of the blood, there exist privileged routes for glucose diffusion to distinct brain regions (e.g., from cerebrospinal fluid to ARC but not VMN) which are regulated by nutritional status. These observations also underscore the putative role of central glucose sensing in modulating glucose levels in order to meet the needs of the brain.

## Cellular mechanisms of glucose sensing

The existence of glucose sensing neurons was suggested by 2 independent groups in 1964, one using cats and the other dogs (Anand et al., 1964; Oomura et al., 1964). In these studies, reciprocal changes in single unit activity were measured in the VMH and lateral hypothalamus (regions formerly called the “satiety” and “feeding” centers, respectively) during intravenous glucose or insulin injections. Glucose increased neuronal activity in the VMH while the converse occurred in the lateral hypothalamus. Later work from Oomura's laboratory demonstrated that hypothalamic neurons were directly regulated by glucose *in vitro*. This group coined the terms *glucose responsive* for neurons that increased their activity with increased glucose and *glucose sensitive* for those that decreased their activity as glucose increased (Oomura et al., 1964). Today, these subtypes of glucose sensing neurons are more commonly referred to respectively as *glucose-excited* (GE) or *glucose-inhibited* (GI) neurons based on their response to physiological changes in extracellular glucose (Song et al., 2001; Kang et al., 2004; Wang et al., 2004; Burdakov et al., 2005; Melnick et al., 2011). The following discussion of glucose sensing mechanism will focus primarily on studies using physiological glucose concentrations unless this information is not available. These data are summarized in Table 1. We are defining physiological glucose concentration as being below 5 mM in order to account for variability in blood or cerebrospinal fluid to brain glucose ratio under differing physiological conditions. Data from studies which used tissue or cellular preparations which do not discriminate between the ARC and VMN will be referred to as “VMH,” whereas studies in which neuronal location was verified visually or via microdissection as being from one region or the other will be referred to as such.

**Table 1:** **Putative role of glucose-inhibited and glucose-excited neurons in substrate partitioning during energy deficit**.

**Nuclei/Peptide phenotype**	**Glucose Sensing phenotype**	**Mechanism of glucose sensing**	**Putative effect on brain fuel supply during energy deficit**	**Mechanism by which brain fuel may be altered**	**Representative references**
ARC NPY	Inhibited	nNOS independent	Increase	Increase food intake	Murphy et al., 2009b; Joly-Amado et al., 2012; Leshan et al., 2012; Warne et al., 2013; Wang et al., 2014
				Decrease energy expenditure	
				Initiate ketogenesis	
ARC GHRH	Inhibited	Unknown	Increase	Caloric mobilization	Stanley et al., 2013
VMN nNOS	Inhibited	NO/AMPK/Cl^−^ channel	Increase	Glucagon, epinephrine secretion	Song et al., 2001; Song and Routh, 2006; Murphy et al., 2009a; Fioramonti et al., 2010a, 2013
LH orexin	Inhibited	Metabolism independent	Increase	Reward feeding Arousal	Antunes et al., 2001; Burdakov et al., 2005; Harris et al., 2005; Gonzalez et al., 2008; Williams et al., 2008; Sheng et al., 2014
		K^+^ channel			
LH NPY	Inhibited	K^+^ channel	?		Marston et al., 2011
LH GABA	Inhibited	Metabolism independent	?	?	Karnani et al., 2013
		Mixed ionic conductance			
PVN pre-autonomic	Inhibited	Unknown	?	?	Melnick et al., 2011
LH MCH	Excited	K^+^ channel	Decrease	Anabolic (increase fat mass)^*^	Burdakov et al., 2005; Naufahu et al., 2013
PO/AHA GnRH	Excited	AMPK	Decrease	Divert fuels to reproductive axis	Roland and Moenter, 2011; Beall et al., 2012
		Non–selective cation channel			
VMN unknown	Excited	KATP channel	?	?	Ashford et al., 1990a; Song et al., 2001
PVN pre-autonomic	Excited	KATP independent	?	?	Melnick et al., 2011
		Non–selective cation channel			

The overall hypothesis presented here is that during energy deficit the glucose-sensing function of these neurons reinforces the mechanisms which provide brain-specific fuels while reducing the drive for other fuel consuming neuroendocrine systems. The predictions regarding the putative effect on brain fuel supply and underlying mechanisms are based on net positive input to downstream circuitry and would be reversed if their net effect was inhibitory.

* While MCH neurons increase food intake, they are associated with anabolic processes which could divert fuel away from the brain.

### Glucose-excited (GE) neurons

GE neurons are found in many hypothalamic regions including the ARC, VMN, anterior hypothalamus, paraventricular nucleus, and the lateral hypothalamus (Oomura et al., 1964; Ashford et al., 1990a,b; Shian and Lin, 1991; Burdakov et al., 2005; Melnick et al., 2011; Roland and Moenter, 2011). The neurotransmitter phenotype of VMN GE neurons is largely unknown and there are conflicting reports regarding whether ARC POMC neurons are GE neurons (Wang et al., 2004; Claret et al., 2007; Fioramonti et al., 2007; Parton et al., 2007). In contrast, GE neurons have been found among the gonadotropin releasing hormone neurons (GnRH) of the anterior hypothalamus (Roland and Moenter, 2011; Beall et al., 2012), the preautonomic neurons in the paraventricular nucleus (Melnick et al., 2011), and the melanin concentrating hormone neurons of the lateral hypothalamus (Burdakov et al., 2005). The mechanism underlying glucose sensing by VMH GE neurons has been the most extensively characterized. Michael Ashford was the first to show that the majority of these GE neurons utilize a glucose sensing mechanism similar to that of the pancreatic β-cell (Ashford et al., 1990a). That is, like the β-cell, increased glucose metabolism raises the ATP/ADP ratio and closes the ATP sensitive potassium channel (KATP) leading to depolarization. Despite the fact that Ashford's original studies were performed using non-physiological glucose concentrations, his findings have stood the test of time (and glucose concentration) (Song et al., 2001). Barry Levin's laboratory demonstrated a second important similarity between many VMH GE neurons and the pancreatic β-cell: the initial phosphorylation of glucose to glucose-6-phosphate is catalyzed by the pancreatic form of hexokinase IV, glucokinase (Dunn-Meynell et al., 2002; Kang et al., 2006). In addition to a lower affinity for glucose than other hexokinases, glucokinase is not subject to end-product inhibition. This allows for activity at a rate proportional to glucose concentration (Magnuson, 1990). Thus, the presence of glucokinase is often used to predict glucose sensing in neuronal populations (Navarro et al., 1996; Yang et al., 1999; Lynch et al., 2000; Maekawa et al., 2000; Mobbs et al., 2001; Dunn-Meynell et al., 2002; Levin, 2002; Penicaud et al., 2002; Ramonet et al., 2004; Balfour et al., 2006; Kang et al., 2006; Zhou et al., 2010; Beall et al., 2012).

In addition to the ubiquitous glucose transporter 3 (GLUT3), subpopulations of VMH GE neurons also express GLUT2 and the insulin-sensitive GLUT4. Interestingly, a fairly large percentage of GE neurons express both GLUT4 and the insulin receptor (Kang et al., 2004). About 25% of VMH GE neurons possess the sodium glucose co-transporter (SGLT) which could couple glucose uptake with membrane depolarization (Kang et al., 2004). The cellular fuel sensor AMP activated protein kinase (AMPK) may mediate glucose sensing in some GE neurons. For example AMPK dependent glucose sensing has been reported for POMC neurons, as well as for the GnRH neurons in the anterior hypothalamus (Claret et al., 2007; Roland and Moenter, 2011; Beall et al., 2012). Finally, the KATP channel does not mediate glucose sensing in all GE neurons. GnRH neurons and PVN GE neurons use a non-selective cation channel, while the melanin-concentrating hormone neurons use an unidentified K channel (Burdakov et al., 2005; Melnick et al., 2011; Roland and Moenter, 2011). Thus, while many GE neurons could be considered the brain's “pancreatic β-cell,” there is clearly a high degree of heterogeneity within this category of glucose sensing neurons.

### Glucose-inhibited (GI) neurons

GI neurons are also widely dispersed throughout the hypothalamus, including the VMN, ARC, paraventricular nucleus, dorsomedial nucleus, and the lateral hypothalamus (Song et al., 2001; Burdakov et al., 2005; Song and Routh, 2006; Fioramonti et al., 2007; Williams et al., 2008; Marston et al., 2011; Melnick et al., 2011). There are two broad categories of GI neurons, those whose activity is regulated by glucose metabolism (e.g., by changes in ATP) and those that respond to the glucose molecule itself. The *metabolism dependent* GI neurons most likely utilize GK since, like GE neurons, the majority of GI neurons express GK (Dunn-Meynell et al., 2002; Kang et al., 2006). Moreover, like GE neurons, subpopulations of metabolism dependent GI neurons also express GLUT2, GLUT4, SGLT2, and/or the insulin receptor (Kang et al., 2004). The most studied of the metabolism dependent GI neurons are located in the VMH. These GI neurons are activated in low glucose via an interaction between AMPK and the gaseous messenger nitric oxide (NO) which leads to chloride channel closure, membrane depolarization, and increased action potential frequency (Song et al., 2001; Song and Routh, 2005; Murphy et al., 2009a; Fioramonti et al., 2010a). VMH NO production by the neuronal NO synthase (nNOS) and NO signaling through its receptor, soluble guanylyl cyclase (sGC) are critical for both glucose sensing by GI neurons and the *in vivo* counterregulatory response (CRR) to hypoglycemia suggesting a role for these GI neurons in hypoglycemia detection (Murphy et al., 2009a; Fioramonti et al., 2010a, 2013). There is also a very significant overlap between VMH NO producing neurons and GI neurons (Canabal et al., 2007b). Thus, while the peptide neurotransmitter phenotype of these GI neurons remains unclear, it is reasonable to refer to them as nNOS-GI neurons based on their gaseous transmitter phenotype.

The above studies provided consistent results using either electrophysiological recordings of VMN GI neurons in brain slices or membrane potential dye imaging in freshly isolated VMH neurons. The electrophysiological experiments were performed on visually identified VMN neurons; whereas studies using isolated VMH neurons did not allow discrimination between VMN and ARC neurons. It is likely that mechanistic data concerning AMPK-NO signaling obtained from imaging isolated VMH neurons reflect VMN vs. ARC GI neurons for several reasons. Technically, VMN neurons vastly outnumber ARC neurons in this preparation and thus the significant majority of GI neurons analyzed would be VMN- vs. ARC-GI neurons. Moreover, the ARC neuropeptide Y (NPY)/agouti-related peptide (AgRP) co-expressing neurons, half of which are GI neurons, do not express nNOS nor do changes in intracellular AMPK appear to affect their activity (Sternson et al., 2005; Fioramonti et al., 2007; Leshan et al., 2012). Thus, together these data suggest distinct glucose sensing mechanisms for VMN nNOS-GI and ARC NPY/AgRP-GI neurons. The exact cellular mechanism underlying glucose sensing in the ARC NPY/AgRP-GI neurons is not known. However, it could still be argued that regardless of what the mechanism is ultimately determined to be, the NPY/AgRP-GI neurons are metabolism-dependent since they are clearly regulated by changes in AMPK activity in presynaptic terminals synapsing onto NPY/AgRP neurons (Yang et al., 2011).

Denis Burdakov's laboratory has extensively characterized the *metabolism-independent*, orexin expressing GI neurons, which are only found in the lateral, perifornical and dorsomedial hypothalamus (Burdakov et al., 2005; Gonzalez et al., 2008; Williams et al., 2008). Orexin neurons are equally inhibited by glucose and by the non-metabolizable glucose analog, 2-deoxyglucose. Thus, they respond to the glucose molecule itself rather than downstream metabolites (Gonzalez et al., 2008; Sheng et al., 2014). Glucose clearly activates a leak tandem-pore potassium (K2P) channel in these neurons (Burdakov et al., 2006; González et al., 2009). Moreover, Twik-related acid-sensitive potassium-like (TASK) 1/3 channels, which belong to the K2P ion channel family, regulate high frequency firing in orexin neurons. However, TASK 1/3 channels do not appear to be critical for glucose sensing (González et al., 2009). Thus, at the present time the precise ion channel which mediates glucose inhibition in metabolism-independent GI neurons remains to be determined. Interestingly, there are two different glucose responses for metabolism-independent GI neurons. A non-adapting response is characterized by sustained glucose inhibition, while “adapting” GI neurons are transiently inhibited when glucose concentration rises but resume firing action potentials after several minutes in high glucose (Williams et al., 2008). The adaptive response appears to be due to a spontaneous decrease in inhibitory K^+^ currents (Williams et al., 2008). In addition to the orexin neurons, GI neurons also exist within the NPY and gamma-aminobutyric acid (GABA) neurons of the lateral hypothalamus (Marston et al., 2011; Karnani et al., 2013). The GABA-GI neurons are also metabolism independent. However, they are distinct from the orexin-GI neurons in that the glucose sensitive conductance appears to be carried by a mixture of ion channels (Marston et al., 2011). It is not known whether the lateral hypothalamic NPY-GI neurons are metabolism dependent or independent (Karnani et al., 2013). The paraventricular nucleus GI neurons may also be metabolism independent because they do not appear to express GK (Stanley et al., 2013).

### Glucose sensing glial cells

Neurons are not the only glucose sensitive cells within the brain. An increasing body of knowledge suggests that glucose sensing by hypothalamic glial cells may play an important role in glucose and energy homeostasis. For example, the data discussed above indicates that the cells composing the blood brain barrier are responsive to changes in glucose (Langlet et al., 2013). Specialized ependymal cells known as tanycytes which line the third ventricle express GK, GLUT2 and KATP channels, suggesting that they are capable of sensing glucose (Garcia et al., 2003). In support of this, glucose increases intracellular calcium levels within cultured tanycytes (Orellana et al., 2012). Hypothalamic GK inhibition by alloxan *in vivo* reduces food intake, impairs the hyperglycemic response to a glucoprivic challenge and destroys tanycytes; all of which reverse 2 weeks after alloxan injection. These data suggest that hypothalamic tanycytes play a role in the physiological response to hypoglycemia (Sanders et al., 2004). Astrocytes also play a role in glucose sensing. Numerous studies suggest that the glucose transporter 2 (GLUT2) plays a role in hypothalamic glucose sensing (Garcia et al., 2003; Guillod-Maximin et al., 2004; Marty et al., 2005; Bady et al., 2006; Stolarczyk et al., 2010). Interestingly, hypothalamic GLUT2 is primarily expressed in astrocytes (Guillod-Maximin et al., 2004). Thus, it is clear that hypothalamic glucose sensing is a complex system involving coordination between a wide variety of neuronal and glial populations.

## Regulation of glucose sensing by signals of peripheral energy homeostasis

### Hormonal signals of energy homeostasis

Hypothalamic glucose sensing is modulated by the hormonal milieu which reflects peripheral energy homeostasis. Insulin and the adipose derived hormone leptin, both of which are considered “satiety” hormones, inhibit VMH GE neurons in the presence of high extracellular glucose by opening the KATP channel (Spanswick et al., 1997, 2000). The effect of insulin persisted in physiological glucose concentrations (Cotero and Routh, 2009); however leptin had no effect on VMH GE neurons in 2.5 mM glucose (Wang et al., 2004). Insulin also prevents inhibition of VMH GE neurons when glucose is lowered (Cotero and Routh, 2009; Cotero et al., 2009). Interestingly, hypothalamic insulin infusion suppresses hepatic glucose production *in vivo* (Rojas and Schwartz, 2014). A role for VMH GE neurons in altered glucose homeostasis is also suggested by the observation that VMH GE neurons from *db/db* mice and *fa/fa* Zucker rats with peripheral insulin resistance are also insulin resistant (Spanswick et al., 2000; Cotero et al., 2009). Interestingly, ghrelin, which is released from the stomach during hunger, indirectly inhibits ARC POMC (GE?) neurons (Cowley et al., 2003; Ibrahim et al., 2003). Similar effects of ghrelin and leptin on GE neurons is counterintuitive. However, it is well established that leptin activates POMC neurons (Cowley et al., 2001). Moreover, the effect of ghrelin on glucose sensitivity of POMC neurons has not yet been evaluated nor is there agreement as to whether POMC neurons are truly GE neurons (Wang et al., 2004; Fioramonti et al., 2007).

Metabolically relevant hormones also regulate both the activity and the glucose sensitivity of GI neurons. Leptin inhibits ARC NPY/AgRP neurons (50% of which are GI neurons) as well as lateral hypothalamic orexin-GI neurons (Shiraishi et al., 2000; Yamanaka et al., 2003; Kohno et al., 2007; Louis et al., 2010; Baver et al., 2014). Leptin also blunts the activation of both VMH- and lateral hypothalamic orexin-GI neurons by decreased glucose (Murphy et al., 2009b; Sheng et al., 2014). In contrast, activation of VMN GI neurons by decreased glucose is blunted in the neuronal insulin-receptor knockout (NIRKO) mouse (Diggs-Andrews et al., 2010) and insulin stimulates NO production by VMH GI neurons in decreased glucose (Canabal et al., 2007b). These observations are consistent with insulin-induced potentiation of the response to low glucose in these neurons. The effect of insulin on lateral hypothalamus orexin-GI neurons is not clear. These neurons are clearly activated by insulin-induced hypoglycemia; however whether this is simply because they are GI neurons or whether insulin is having an independent effect is not known (Griffond et al., 1999; Moriguchi et al., 1999; Cai et al., 2001; Zhao et al., 2005). As predicted given the inhibitory effect of leptin, ghrelin activates both NPY-GI and orexin-GI neurons (Kohno et al., 2003; Yamanaka et al., 2003). Ghrelin also increases the activation of orexin-GI neurons in low glucose (Sheng et al., 2014).

### Nutrients other than glucose

Hypothalamic glucose sensing neurons also respond to other nutrient-related signals in addition to glucose. Within the VMH, there is overlap between GE and GI neurons and neurons whose activity is altered by free fatty acids and ketones (Wang et al., 2005; Le Foll et al., 2013, 2014). Neuronal fatty acid sensing is consistent with data showing that central fatty acid infusion modulates glucose-induced insulin secretion and peripheral insulin sensitivity (Cruciani-Guglielmacci et al., 2004; Migrenne et al., 2006; Marsollier et al., 2009; Le Foll et al., 2013). In contrast to glucose and fatty acids, direct evidence of amino acid sensing neurons has been elusive. Administration of the branched chain amino acid L-leucine, but not valine, into the third ventricle decreases food intake; an effect which appears to be mediated by the hypothalamic mammalian target of rapamycin (mTOR) (Cota et al., 2006). The mTOR inhibitor rapamycin restores NO production by VMH GI neurons during hyperglycemia suggesting potential overlap in glucose and amino acid sensing pathways in these neurons (Canabal et al., 2007a). Moreover, Blouet et al. demonstrated that direct application of leucine increased the action potential frequency of POMC neurons (Blouet et al., 2009). Taken together these data support the hypothesis that hypothalamic glucose sensing neurons integrate multiple nutrient signals; however this has yet to be definitely shown for amino acids.

## Physiological role of glucose sensing neurons

While the existence of glucose sensing neurons has been known for half a century (Anand et al., 1964; Oomura et al., 1964), their exact physiological functions remain elusive. In the following discussion, we have attempted to relate what is known about the different subpopulations of glucose sensing neurons with the function of the region or peptide phenotype in which they are found. Table 1 summarizes these data. Our overall hypothesis is that glucose sensitivity within multiple populations of neurons involved in metabolic control enables the brain to broadly reinforce the neurocircuitry which compensates for energy deficit when glucose levels fall. This would be beneficial in order to ensure an adequate neuronal fuel supply (glucose, ketones) at all times.

### Hypoglycemia detection

When considering a physiological role for glucose sensing neurons, hypoglycemia detection seems an obvious choice, especially since the majority of glucose sensing neurons are most sensitive to a decline in extracellular glucose levels below that which would be seen during normal fed conditions (Song et al., 2001; Wang et al., 2004; Song and Routh, 2005; Murphy et al., 2009b). This hypothesis is also attractive when considering that glucose is the primary fuel of the brain and that hypoglycemia clearly has deleterious central effects (Languren et al., 2013). Insulin-induced hypoglycemia increases *cfos* activation in a number of hypothalamic nuclei, including the ARC, the paraventricular and dorsomedial nuclei and the lateral hypothalamus (Niimi et al., 1995; Diggs-Andrews et al., 2010; Fergani et al., 2014). Moreover, hypoglycemia or central glucoprivation induces *cfos* expression in several known hypothalamic glucose sensing populations including the lateral hypothalamic orexin neurons as well as NPY neurons in the ARC, dorsomedial nucleus and lateral hypothalamus (Cai et al., 2001; Ferenczi et al., 2010; Marston et al., 2011). Interestingly, although the VMN contains GI neurons and plays a role in hypoglycemia detection, *cfos* activation was not detected in this brain region following insulin-induced hypoglycemia or glucoprivation (Niimi et al., 1995; Borg et al., 1997; Diggs-Andrews et al., 2010; Zhu et al., 2010; Szepietowska et al., 2011; Stanley et al., 2013). One reason for this apparent discrepancy may be because activation of VMN nNOS-GI neurons by decreased glucose requires the NO-sGC pathway, which may not be represented by *cfos* activation.

Although VMN *cfos* does not increase after hypoglycemia, functional studies support a key role for VMN GI neurons in the CRR. For example, repetitive hypoglycemic episodes shift the glycemic threshold for initiation of the CRR to lower glucose levels (Dagogo-Jack et al., 1993; Cryer, 2013; Moheet et al., 2014). Similarly, after recurrent hypoglycemia VMN GE and GI neurons show blunted inhibition or activation, respectively, as glucose levels decline (Song and Routh, 2006; Kang et al., 2008; Fioramonti et al., 2010b). Moreover, VMH lactate infusion *in vivo* blunts the CRR while lactate perfusion *in vitro* blunts activation of VMN GI neurons by decreased glucose (Song and Routh, 2005). VMH NO signaling is critical for glucose sensing by VMN nNOS-GI neurons as well as for the full CRR. That is, nNOS inhibition prevents activation of VMN nNOS-GI neurons by decreased glucose and neurons which are activated by decreased glucose are not detectable in brain sections from nNOS knockout mice (Fioramonti et al., 2010a). Similarly, the CRR is significantly impaired in nNOS knockout mice, after VMH NOS or sGC inhibition, or when NO-sGC signaling is disrupted by a post-translational modification of sGC known as S-nitrosation which renders it NO resistant (Fioramonti et al., 2010a, 2013). Interestingly, when S-nitrosation during recurrent hypoglycemia is prevented both glucose sensing by VMH GI neurons as well as the CRR are preserved. Taken together, these data suggest a clear role for GI, and in particular VMN nNOS-GI, neurons in detecting hypoglycemia and initiating the CRR.

### Fasting and starvation

In addition to hypoglycemia detection, another potential role for glucose sensing neurons is to maintain the brain's energy supply during starvation. In fact, since insulin-induced hypoglycemia is an iatrogenic consequence of treating Type 1 and advanced Type 2 Diabetes Mellitus in the modern world, glucose sensing neurons probably evolved as a defense against starvation. During starvation, the hypothalamic-pituitary-adrenal axis and the sympathetic nervous system play a key role in energy mobilization (Straub et al., 2010). The energetic needs of the body and the brain are first supplied by mobilization of liver glycogen stores for gluconeogenesis. After several hours adipocyte lipolysis provides fatty acids, which then fuel most organ systems. However, the brain cannot utilize fatty acids as fuel. Thus, preserving adequate blood glucose for the brain during starvation is one of the most important actions of the neuroendocrine system. When liver glycogen is depleted, muscle catabolism is initially necessary to provide amino acids as substrates for gluconeogenesis. Muscle catabolism is clearly detrimental in the long term and after several days of starvation, increased conversion of fatty acids to ketones in the liver provides fuel to the brain and spares lean body mass (Straub et al., 2010). It seems likely that glucose sensing neurons, particularly GI neurons, are important for informing the brain of the potential magnitude of a threat to the brain energy supply during starvation and aiding in energy mobilization.

In support of this, in general the hormonal changes which occur during fasting (e.g., reduced leptin and increased ghrelin) activate GI neurons and/or enhance their activation by decreased glucose (Cai et al., 1999; López et al., 2000; Diano et al., 2003; Kohno et al., 2003, 2008; Yamanaka et al., 2003; Murphy et al., 2009b; Sheng et al., 2014). A role for ARC NPY/AgRP-GI neurons in defending the brains energy supply during starvation stands out in particular. Fasting increases NPY gene expression as well as *cfos* expression and action potential frequency in ARC NPY/AgRP-GI neurons (Mizuno et al., 1999; Takahashi and Cone, 2005; Becskei et al., 2009; Murphy et al., 2009b). Fasting also increases hypothalamic NPY release and the activation of NPY/AgRP-GI neurons in response to decreased glucose (Murphy et al., 2009b). These data are consistent with the overall role of hypothalamic NPY/AgRP neurons to stimulate food intake (Billington and Levine, 1992; White, 1993). NPY expressing neurons are found in several brain regions which regulate energy homeostasis including the ARC and brainstem. However, AgRP expression is restricted to the ARC where it is found in 95% of the NPY neurons (Broberger et al., 1998). AgRP is an endogenous inhibitor of the melanocortin receptor. Thus, NPY/AgRP neurons oppose the effects of the anorexigenic POMC neurons at their targets. These targets include the hypothalamic paraventricular nucleus; a key site for regulating metabolism and food intake. The corticotrophin releasing hormone neurons which drive the hypothalamic-pituitary-adrenal axis as well as pre-autonomic neurons which activate the sympathetic nervous system reside in the paraventricular nucleus (Steffens et al., 1972, 1988; van Dijk et al., 1994; Ahima et al., 2000; Elmquist, 2001; Watts and Sanchez-Watts, 2002). In addition to opposing POMC neurons, NPY/AgRP neurons regulate energy partitioning and substrate utilization in a melanocortin-independent fashion (Joly-Amado et al., 2012). Importantly, AgRP deficiency impairs ketone body release from the liver during starvation (Warne et al., 2013). AgRP also mediates the gluconeogenic effects of ghrelin (Wang et al., 2014). Thus, increased AgRP release, especially in response to low glucose during starvation, may play a role in enhancing gluconeogenesis as well as triggering increased ketone production to fuel the brain. Similarly, increased activation of VMN nNOS-GI neurons during low glucose is consistent with enhanced gluconeogenesis (Fioramonti et al., 2010a). Orexin is associated with enhanced reward-based feeding behavior as well as increased sympathetic activity. Increased activity in low glucose, especially during fasting, is consistent with this line of reasoning (Antunes et al., 2001; Harris et al., 2005; Kelley et al., 2005; Zheng et al., 2007; Choi et al., 2010; Perello et al., 2010; Sheng et al., 2014). Moreover, we have recently shown that low glucose enhances excitatory glutamate neurotransmission onto ventral tegmental area dopamine neurons in an orexin dependent manner (Sheng et al., 2014). This observation strongly supports a role for orexin-GI neurons in reinforcing feeding behavior when glucose levels decline. Finally, since growth hormone is also released during hypoglycemia, it makes sense that growth hormone releasing hormone neurons are also GI neurons (Stanley et al., 2013). Together these data are consistent with an overall role for hypothalamic GI neurons in providing the brain with fuel during energy deficit. An important caveat to the above discussion is that the neurocircuitry which controls autonomic and neuroendocrine systems is extremely complex and its activation should never be considered in “all or none” terms. Rather, the above is a generalization in order to propose a physiological role for glucose sensing neurons and form a framework for designing experiments which test these hypotheses.

### Link neuroendocrine systems with glucose status

Another putative function for hypothalamic glucose sensing neurons is to prevent activation of neuroendocrine systems which use rather than provide fuel during energy deficit or conversely to allow energy to be diverted to these systems when it will not threaten the brain. For example, this may explain why subpopulations GnRH neurons are glucose sensing (Roland and Moenter, 2011). In contrast to the GI neurons described above, GnRH neurons are excited by glucose. Thus, when glucose levels are sufficient reproductive function is supported. Conversely, during times when food is scarce and glucose levels are low, the glucose sensing function of these neurons would contribute to inhibition of the reproductive axis. Moreover, while melanocortin concentrating hormone neurons stimulate food intake, they are also associated with anabolic processes (increased fat mass, reproduction) which could divert fuel away from the brain (Naufahu et al., 2013). Thus, reduced activity of melanocortin concentration hormone-GE neurons in low glucose is consistent with our overall hypothesis. However, one must use caution when trying to apply this reasoning to all GE or GI neurons because the relationship between the direction of glucose sensing (i.e., excited or inhibited by glucose) will depend on whether that particular glucose sensing neuron is inhibitory or stimulatory to the system in question. This is further evidenced by the observation that both GE and GI neurons are often found in the same hypothalamic nuclei. As mentioned above, the homeostatic mechanisms regulated by the hypothalamus are diverse and extremely complex. Thus, extreme caution is warranted when attempting to generalize the function of hypothalamic GE and GI neurons. Moreover, it is important to realize that there will be numerous exceptions to any rule for these systems.

### What about energy surplus?

The preceding discussion of hypothalamic glucose sensing neurons has focused on their role in sensing energy deficit. The main reasons for this bias are as follows. Although glucose concentration-response curves have only been published for VMH GE and GI neurons, these data suggest that glucose sensing neurons are primarily responsive to decreases in glucose below brain glucose concentrations associated with peripheral euglycemia (Wang et al., 2004; Song and Routh, 2005; Kang et al., 2006; Murphy et al., 2009b). In addition, their glucose sensitivity is altered under conditions associated with a need to sense energy deficit. That is, fasting reduces the inhibitory effect of glucose on VMH GI neurons leading to increased activation in response to smaller declines in glucose (Murphy et al., 2009b). Moreover, the glucose sensitivity of both VMH GE and GI neurons shifts in parallel with the ability of the brain to sense and respond to insulin-induced hypoglycemia (Song and Routh, 2005, 2006; Kang et al., 2008; Diggs-Andrews et al., 2010; Fioramonti et al., 2010a,b, 2013). In contrast, there is less known about glucose sensing neurons during hyperglycemia. We found that acute hyperglycemia prevents VMH GI neurons from producing NO and depolarizing in response to decreased glucose as a result of mTOR inhibition of AMPK (Canabal et al., 2007a). Conversely, while inhibition of VMH GE neurons from diabetic *db/db* mice to a maximal glucose decrease to 0.1 mM was identical to that of non-diabetic controls, the inhibition in response to a mid-range decrease (0.5 mM) was significantly enhanced in the diabetic mice (Cotero et al., 2009). Spanswick and colleagues found no difference in glucose sensitivity of VMH GE neurons in Zucker obese *fa/fa* rats compared to lean *Fa/Fa* rats; however they only evaluated the response of these neurons to a glucose decrease from 10 to 0 mM (Spanswick et al., 2000). Finally, 2 additional subtypes of ARC glucose sensing neurons have been identified which respond to changes in extracellular glucose above 5 mM: the high-GE (HGE) and high-GI (HGI) neurons (Fioramonti et al., 2004). As mentioned above, brain glucose concentrations above 5 mM have never been observed. However, since the permeability of the blood brain barrier changes with nutrient status it is possible that these glucose sensing neurons may play a role in glucose detection under some situations (Langlet et al., 2013). Such a role is consistent with data from Luc Penicaud's group showing that centrally restricted glucose infusion via the carotid artery increased *cfos* expression in ARC and paraventricular nucleus neurons and astrocytes which was associated with transient peripheral insulin secretion (Guillod-Maximin et al., 2004). Based on the above findings, it is tempting to speculate that the brain has a mechanism for sensing hyperglycemia and modulating peripheral metabolism in response. Furthermore, disrupted glucose sensing in diabetes suggests that glucose sensing neurons may play a role in the pathology of metabolic syndrome. In fact, an enhanced response to glucose deficit during conditions of energy sufficiency or even surplus (e.g., obesity/diabetes) could potentially lead to similar compensatory mechanisms as in response to fasting or starvation. Such changes could further enhance the obese state. However, due to a lack of data such comments are purely speculative at this time.

## Interaction with non-hypothalamic glucose sensors

While the focus of this review is on hypothalamic glucose sensing neurons, glucose sensing neurons are also found in many other brain regions including the amygdala (Nakano and Oomura, 1986; Zhou et al., 2010), nucleus solitarius (Adachi et al., 1984) and midbrain (Levin, 2000). Moreover, although glucose sensing neurons *per se* have not been identified in the hindbrain, neurons within this region clearly respond to glucoprivation (Ritter et al., 1998, 2011; Routh et al., 2013). There are also glucose sensors located in numerous peripheral sites including the carotid bodies (Alvarez-Buylla and de Alvarez-Buylla, 1998; Pardal and Lopez-Barneo, 2002; Lopez-Barneo, 2003), portal-mesenteric vein (Donovan et al., 1994; Hevener et al., 1997; Matveyenko and Donovan, 2006) and intestine (Sayegh et al., 2004; Raybould, 2007; Ackroff et al., 2010). The regulation of glucose homeostasis clearly involves integration of information from multiple glucose sensing regions. For example, the hypothalamus, hindbrain and portal-mesenteric vein glucose sensors are all critical for hypoglycemia detection and full initiation of the CRR (Routh et al., 2013). Unraveling the physiological function of the many glucose sensing brain regions and how they work together is an evolving field which requires a great deal more work in order to understand.

## Conclusion: the selfish brain

In conclusion, the hypothalamus is the primary site of integration for the neuroendocrine and autonomic responses which maintain energy and glucose homeostasis. Within the hypothalamus sensory neurons respond to circulating nutrients and hormones in order to inform the brain regarding peripheral metabolic homeostasis. The presence of glucose sensing neurons within the hypothalamus suggests a sensitive mechanism for maintaining a constant glucose supply to the brain. There is evidence for a role of glucose sensing neurons in hypoglycemia detection and initiation of the CRR during modern iatrogenic hypoglycemia (Song and Routh, 2006; Fioramonti et al., 2010a,b, 2013; Routh et al., 2013). However, it is likely that glucose sensing neurons actually evolved to protect the brain during intermittent starvation. For example, fasting increases the activation of NPY/AgRP-GI and orexin-GI neurons by decreased glucose (Murphy et al., 2009b). This could provide glucose for the brain in several ways; including increased food intake, initiating gluconeogenesis and mediating the shift to ketogenesis should the threat be sufficient (Beck, 2000; Joly-Amado et al., 2012; Warne et al., 2013; Sheng et al., 2014; Wang et al., 2014). Should similar fasting-induced changes in the glucose sensitivity of VMN nNOS- and growth hormone releasing hormone-GI neurons occur, this could also contribute to ensuring adequate fuel to the brain (Cai et al., 1999; Fioramonti et al., 2010a; Stanley et al., 2013). In contrast, other subtypes of glucose sensing neurons may divert energy away from competing systems (e.g., reproductive axis Roland and Moenter, 2011) when glucose levels are low. On the other hand, when the energy needs of the brain are met, the response of glucose sensing neurons to low glucose may be “deactivated” as seen for the effect of leptin and insulin on VMH GI and GE neurons, respectively (Cotero and Routh, 2009; Murphy et al., 2009b). Thus, we hypothesize that during energy deficit glucose sensing neurons are enlisted in order to enable the brain to prioritize its own energy needs at the expense of other energy requiring systems. However, clearly much work remains to determine the viability of our hypothesis.

## Author contributions

VHR provided the original intellectual conception for the work and drafted and revised the manuscript. Lihong Hao, Ammy M. Santiago, Zhenyu Sheng and Chunxue Zhou provided intellectual discussion during conception and provided critical revision of the draft. All authors provide final approval and agree to be accountable for all aspects of the work in ensuring that questions related to the accuracy or integrity of any part of the work are appropriately investigated and resolved.

### Conflict of interest statement

The authors declare that the research was conducted in the absence of any commercial or financial relationships that could be construed as a potential conflict of interest.
